# Towards a genetic AIDS vaccine

**DOI:** 10.1186/1742-4690-6-93

**Published:** 2009-10-16

**Authors:** Antonia V Bordería, Ben Berkhout

**Affiliations:** 1 Laboratory of Experimental Virology, Department of Medical Microbiology, Center for Infectious Diseases and Immunology Amsterdam (CINIMA), Academic Medical Center (AMC), University of Amsterdam, the Netherlands

## Abstract

We discuss a recent Nature Medicine publication by Philip Johnson and co-workers (Vector-mediated gene transfer engenders long-lived neutralizing activity and protection against SIV infection in monkeys. *Nat. Med*. 2009, 15: 901-906) in which an effective HIV-1 vaccine was designed that is based on gene therapy. The introduced gene produces an antibody-like immunoadhesin in the blood that neutralizes the virus.

## Commentary

### The current status of HIV vaccines

The quest for a vaccine against the human immunodeficiency virus type 1 (HIV-1) has recently been given a bad prognosis [1]. Most attempts at developing an HIV-1 vaccine have used substances (immunogens) aimed at stimulating the body's own immune system to produce antibodies or killer T cells that would either neutralize the virus during transmission from one to the other host (protective vaccine) or control the level of virus replication in an individual who is already infected by HIV-1 (therapeutic vaccine). Such traditional HIV vaccines have not elicited protective immune responses, and it is apparent that out-of-the-box thinking is needed to generate some truly innovative ideas that should help us in this HIV-1 vaccine crisis.

### A genetic twist to HIV-1 vaccines

The study by Philip Johnson and his colleagues of the Children's Hospital in Pennsylvania published in a recent issue of Nature Medicine presents an unorthodox, yet surprisingly simple approach. These authors used gene transfer technology to produce antibody-like molecules in the blood that effectively block viral infection [2]. First, they created artificial antibody-like proteins called immunoadhesins that were specifically designed to bind to the simian immunodeficiency virus (SIV) that infects macaques to cause an AIDS-like disease. Second, a gene therapy approach was used to deliver the antiviral immunoadhesin gene into the macaques. This immunoadhesin gene therapy approach bypasses the immune system altogether, and promising results were reported in the pre-clinical macaque model. The insights gained from how to achieve protection against SIV infection by a gene therapy approach could possibly be translated to the control of HIV-1 infection in humans.

### Antiviral immunoadhesins

The antibody genes chosen in the study are immunoadhesins, artificial chimeric antibody-like molecules that were pre-selected to bind the Envelope protein (Env gp120) on the SIV virions. The immunoadhesins are based on two previously characterized gp120-specific Fab clones from SIV-infected macaques. These Fabs show potent *in vitro *neutralizing capacity against the SIVmac316 isolate, and also against other SIV strains. The immunoadhesins were designed by coupling the variable heavy (V_H_) and variable light (V_L_) chains from the Fabs via a linker to an IgG2 Fc fragment of the rhesus macaque (constructs 4L6 and 5L7). In a separate construct N4, the IgG2 domain was fused to the rhesus macaque CD4 molecule (domain 1 and 2), the primary receptor for SIV entry into cells. The purpose of both the Fab and the CD4 molecules is to block the viral Envelope protein and thus abrogate the capability of the virus to bind and infect target cells. Based on the binding affinity, it could be expected that CD4 would be less efficient in virus neutralization than the Fabs. In addition, the CD4 molecules may cause unwanted side effects through interaction with other molecules of the immune system, e.g. the T cell receptor complex.

### AAV gene therapy vector

A harmless adeno-associated virus (AAV) vector was used to deliver the antiviral immunoadhesin or CD4 gene. The AAV vector has some clear advantages over the traditional use of adenoviruses or other vector systems in gene therapy. First, AAV has a relatively low immunogenicity and only triggers the synthesis of neutralizing antibodies, but no cytotoxic T cell activity. Second, AAV vectors can provide a durable therapeutic effect because of the high transduction efficiency and the accumulation of episomal concatamers. A disadvantage of AAV vectors is the limited cloning capacity, which complicates the usage of large genes. The traditional AAV vector was used for insertion of the CD4 gene. As pointed out by the authors, by cloning the expression cassettes between the AAV inverted terminal repeats (ITR), two genomes can anneal to double the capacity of the vector. This novel vector arrangement was used for the immunoadhesin genes. The authors confirmed that the designed immunoadhesin-IgG2 genes were functionally expressed. Initial tests were performed in mice [3], and based on promising results, the authors moved to the macaque model.

### Macaque immunization

Three groups of 3 rhesus macaques were immunized by intramuscular AAV injection. The animals received either the 4L6 or 5L7 vector encoding an anti-SIV immunoadhesin or the N4 vector that encodes the rhesus macaque CD4 molecule. All macaques showed a detectable level of transgene protein expression in the sera. The immunoadhesin concentration in the blood was similarly high for the 4L6 and 5L7 vectors, and persisted for over a year. A somewhat reduced protein level was measured for the N4 vector, which could be due to differences in the AAV vector used. We note that both the AAV vector and the SIVmac316 challenge virus stock were prepared in the human 293-HEK cell line, which may cause cross-species reactions that were not addressed in the study design that used unvaccinated control animals.

### The SIV challenge experiment

The 9 immunized macaques and 6 control non-immunized macaques were challenged one month after immunization by intravenous injection of the live, AIDS-causing SIVmac316 strain. This challenge was repeated to ensure virus transmission, and indeed all control macaques became infected. Four of these control animals had to be sacrificed due to AIDS-related complications, and only a single animal was able to suppress spontaneously viral replication. In contrast, 6 of the 9 immunized macaques remained uninfected after challenge. Furthermore, none of the 9 immunized animals suffered any AIDS-related complications for up to 85 weeks. The protection induced by the immunoadhesin expressing 4L6 vector seemed most effective (3 of 3 animals tested), followed by the CD4-IgG2 expressing vector N4. The single 5L7 animal that was protected from infection showed a pattern of immunoadhesin abundance and neutralization activity similar to the 4L6 monkeys.

All control macaques developed SIV-specific gp120 and Gag antibodies due to the successful infection. The 3 immunized macaques that appeared not to be protected against SIV challenge also developed antibodies against gp120, which could in fact be distinguished from the anti-gp120 immunadhesin expressed from the AAV vector. The 6 protected macaques exhibited different neutralization patterns. The 4L6 and 5L7 groups showed a higher level of neutralizing titers than the N4 group. The macaques that failed to be protected by AAV-immunization, 2 animals in the 5L7 group and 1 in the N4 group, showed a strong immunoadhesin-specific antibody response in comparison with the protected animals that did not develop any immune reaction against the immunoadhesins. The positive role of the IgG2 Fc domain in the effector functions of the immunoadhesin warrants further studies.

### Towards an HIV-1 vaccine

Based on the important proof of concept in the SIV-macaque model, the Johnson team is gearing up for clinical trials with potential "superantibodies" from people who spontaneously resist HIV-1 infection. At the same time, the authors cautioned that many hurdles remain before the technique used in this animal study might be translated into an HIV-1 vaccine for humans. The approach is still in the early stages of animal testing, and there is much to do in order to prove that this approach could yield a successful and safe vaccine that protects against HIV-1 infection. For instance, the monkeys were challenged by direct SIV injection in the blood, exactly the location where the antiviral immunoadhesins reside. Additional tests will be needed to show that the vaccine also protects against sexually acquired virus, as the virus may not encounter the antiviral immunoadhesins in this natural transmission route across the mucosa during the first days of virus replication.

Any test of vector-mediated delivery of protective antibody genes will require extensive tests with a range of antibodies and subsequent optimization tests before human safety studies can take place. For instance, the best protection was observed in those animals that did not mount an immune response against the immunoadhesin vaccine. This finding would argue for the development of improved "humanized" versions of the vaccine so that the human body does not produce vaccine-inactivating antibodies, which then would also allow repeated treatments as booster vaccinations. The final choice of antibody will depend on its ability to protect against the different HIV-1 subtypes. A combination of antibodies could be developed to widen the breadth of the antiviral response. Alternatively, it may be better to develop several subtype-specific gene vaccines that can be used in matching geographic regions, e.g. subtype A in West Africa and subtype C in East Africa. Repeated and massive viral challenges should be done in long-term follow-up experiments to test the longevity of protection and to study the potential for the evolution of vaccine-escape virus variants that could theoretically start to spread in an unrestricted manner in the "gene vaccine protected" population.

Such an unorthodox gene-based vaccine will likely be tested first in small groups of volunteers with a high risk of infection, e.g. discordant couples where one partner is HIV-positive and the other could be vaccinated. As the proposed vaccine will not likely be given to the general population, vector production issues seem less of an urgent issue. These issues would include the stability of the AAV vectors and the ability to manufacture large-scale quantities of these vectors in a cost-effective manner. Finally, this gene vaccination technology may also have potential use in the prevention of other infectious diseases that resist orthodox vaccination strategies.

### Alternative vaccine approaches

One could argue that the only hope of controlling the HIV pandemic is to develop a protective vaccine. The urgency to do so has not really been tempered. HIV-1 has killed 25 million people since the early 1980s and currently infects 33 million individuals. Even if the gene therapy technique leads to an effective HIV vaccine, such a vaccine may be years away from practical application. Thus, it seems wise to develop more unconventional vaccination strategies in order to spread our chances on winning the battle against HIV-1. This includes the use of replication-competent viral delivery systems [4] and possibly even the use of safe versions of a live-attenuated HIV-1 vaccine [5]. In addition, one should speed up approaches to remodel the viral Env protein such that it becomes an effective immunogen that elicits neutralizing antibodies [6]. It cannot be predicted which method will turn out to be the best in terms of vaccine efficacy and breadth of protection. A renewed focus on live-attenuated virus variants seems warranted to define the correlates of protection, a pivotal issue that cannot be learned from the premade immunoadhesin strategy.

### Alternative gene therapy approaches

Future gene therapy has been proposed for individuals who are already infected by HIV-1. Several gene therapy trials are ongoing with antiviral molecules such as ribozymes [7] or inhibitors based on the mechanism of RNA interference (RNAi) [8]. The RNAi approaches have yielded especially impressive results when targeting the viral RNA genome [9], but one could also think of targeting an important cellular co-factor. The CCR5 receptor is an obvious and attractive target. HIV-1 infected people who carry a defective CCR5 gene, CCR5-Δ32, show delayed disease progression, and people homozygous for CCR5-Δ32 are healthy and largely protected from HIV-1 infection [10]. Targeting of CCR5, resulting in perhaps only a partial knock-down, is expected to provide a therapeutic benefit for HIV-infected patients. This potential was recently highlighted in a proof of principle study in non-human primates [11]. Primates received blood stem cells treated with an SIV lentiviral vector expressing a shRNA against CCR5. CCR5 expression was knocked down and T cells from these primates were found to be less susceptible to SIV infection as compared to the appropriate control cells. The primates exhibited normal hematopoietic reconstitution, an important indication that the treatment is safe.

The potential of such an anti-CCR5 gene therapy is further supported by the functional cure of an HIV-infected patient, who had leukemia in addition to AIDS, and received a special bone marrow transplant [12]. The patient's medical condition warranted the high risk blood stem cell transplant, although 10% to 30% of people who receive bone marrow transplants die. Even though the odds are extremely small, a donor was identified who was both a good tissue match and a carrier of the CCR5-inactivating mutation. Prior to the transplant, a standard regimen of drugs and radiation was administered to kill the patient's bone marrow cells and many cells of the immune system. Antiretroviral treatment was stopped when the donor cells were transfused because of concerns about their survival ability. While the plan was to resume the antiretroviral regimen once HIV-1 re-emerged in the patient's blood, standard tests have not detected HIV-1 in his blood for more than 600 days. As stated above, this treatment is unthinkable for the millions of people living with HIV/AIDS. Nevertheless, the results form a "virtual proof of principle" for the safety and efficacy of CCR5-targeting gene therapy approaches in humans.
